# L‐Carnosine Enhances the Proliferation and Myogenic Differentiation of Yanbian Cattle Skeletal Muscle Satellite Cells for Cultured Meat Production via Activating the Akt/mTOR/P70S6K Signaling Pathway

**DOI:** 10.1002/fsn3.71784

**Published:** 2026-04-24

**Authors:** Bin Sun, Huaina Jin, Xuanying Xin, Sajida Naseem, Seong‐Ho Choi, Xiangzi Li, Sungkwon Park

**Affiliations:** ^1^ Engineering Research Center of North‐East Cold Region Beef Cattle Science and Technology Innovation, Ministry of Education Yanbian University Yanji China; ^2^ Department of Animal Science Chungbuk National University Cheongju Korea; ^3^ Department of Food Science and Biotechnology Sejong University Seoul Korea

**Keywords:** Akt/mTOR/P70S6K signaling pathway, antioxidant activity, L‐carnosine, proliferation and differentiation, skeletal muscle satellite cells, yanbian cattle

## Abstract

The composition of muscle fiber types and the development of skeletal muscle are critical determinants of cultured meat quality. L‐carnosine, a dipeptide abundant in ruminant muscle, is known to influence meat quality, yet its regulatory mechanisms in bovine skeletal muscle satellite cells (BSCs) for cultured meat production remain unclear. This study aimed to elucidate the effects of L‐carnosine on the proliferation, differentiation, and muscle fiber type transformation of Yanbian cattle BSCs. We identified 10 mM as the optimal concentration for enhancing cell proliferation (*p* < 0.05), a key finding established by screening L‐carnosine treatments from 0 to 40 mm. This enhancement was mediated by the upregulation of cell cycle genes (Pax7, Ki67, CDK1, CDK2, PCNA) and the suppression of inhibitors (*p21, p53, p16*). Furthermore, L‐carnosine robustly promoted myotube formation and specifically upregulated fast‐twitch muscle fiber markers (MyHC2a, MyHC2b, MyHC2x) while downregulating the slow‐twitch marker MyHC1 (*p* < 0.05). Transcriptomic analysis identified 449 differentially expressed genes, which were significantly enriched in the PI3K‐Akt signaling pathway. Western blotting confirmed that L‐carnosine activates the Akt/mTOR/P70S6K signaling pathway to drive myogenesis. Additionally, L‐carnosine demonstrated significant antioxidant capacity by reducing reactive oxygen species (ROS) and lipid peroxidation (MDA) while enhancing antioxidant enzyme activities (SOD and GSH‐Px). In conclusion, this study provides the first evidence that L‐carnosine promotes BSC proliferation and fast‐twitch fiber differentiation via the Akt/mTOR/P70S6K pathway, suggesting its potential as a highly effective, natural additive for cultured meat production.

## Graphical abstract

This study demonstrates that L‐Carnosine promotes the proliferation and myogenic differentiation of Yanbian cattle skeletal muscle satellite cells by activating the Akt/mTOR/P70S6K signaling pathway, providing a potential strategy for enhancing cultured meat production.

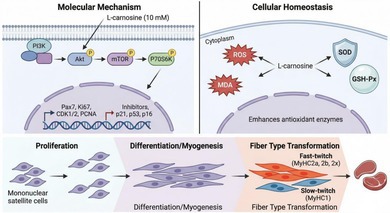

## 1 Introduction

The growing demand for sustainable and ethical alternatives to traditional livestock farming has sparked significant interest in cultured meat production. Cultured meat, produced through the in vitro culture of animal cells, offers a promising solution to address the environmental, ethical, and health concerns associated with conventional meat production. One of the key challenges in cultured meat production is optimizing the proliferation and differentiation of skeletal muscle cells, particularly satellite cells, which are the precursor cells responsible for muscle growth. (Tan and Jiang 2024; Yu et al. 2024). Skeletal muscle, as the primary component of beef, directly determines the final meat quality through its tissue structure, metabolic characteristics, and developmental patterns. Satellite cells in skeletal muscle, as the only population of adult muscle stem cells, are responsible for muscle growth, maintenance, and regeneration postnatally. Under normal physiological conditions, satellite cells remain in a quiescent state and express high levels of Pax7 to maintain their stemness. Upon growth stimuli, they are rapidly activated and enter the proliferative phase, upregulating the expression of cell cycle‐related genes, including CDK1, CDK2, and PCNA (Lee et al. 2023; Ma et al. 2023). Subsequently, driven by MyoD, they initiate myogenic differentiation, sequentially expressing muscle‐specific markers such as MyoG and MyHC, ultimately fusing to form multinucleated myotubes (Lee et al. 2023; Zhao et al. 2024). This dynamic balance between proliferation and differentiation directly influences the number, size, and composition of muscle fibers. Studies have shown that muscle fibers can be classified into four types based on the isoform of myosin heavy chain (MyHC): MyHC I (slow oxidative, type I fibers), MyHC IIa (fast oxidative, type IIa fibers), MyHC IIx (intermediate, type IIx fibers), and MyHC IIb (fast glycolytic, type IIb fibers). Different muscle fiber types exhibit significant differences in metabolic characteristics, contraction speed, myoglobin content, and antioxidant enzyme activity, which in turn influence meat quality parameters such as tenderness, color, pH, and water‐holding capacity (Mo et al. 2023; Wang et al. 2024). Research has shown that appropriately increasing the proportion of fast‐twitch fibers, particularly type IIa fibers, can improve meat tenderness and flavor. Although the number of skeletal muscle fibers is largely fixed at birth, their structure and function exhibit substantial plasticity, allowing for interconversion between type I and type II fibers under certain conditions. Furthermore, oxidative stress, a key factor influencing satellite cell function, can lead to cell cycle arrest, DNA damage, and even apoptosis due to excessive accumulation of reactive oxygen species. Therefore, identifying bioactive factors that can simultaneously promote satellite cell proliferation and differentiation while maintaining their antioxidant capacity is crucial for improving beef quality (Lyu et al. 2024; Wen et al. 2022). L‐carnosine, an endogenous dipeptide composed of β‐alanine and L‐histidine, is one of the most abundant histidine dipeptides in skeletal muscle, with particularly high concentrations in the skeletal muscle tissues of ruminants. As a multifunctional bioactive molecule, L‐carnosine not only exhibits significant pH buffering capacity, which can delay muscle fiber acidification during high‐intensity exercise or metabolic acidosis, but also plays a crucial role in oxidative stress resistance and maintaining cellular metabolic homeostasis through its metal chelation and free radical scavenging properties (Hajimoradi et al. 2023; Hipkiss 2018; Marcotte et al. 2015; Sale et al. 2013). Currently, the industrial scale‐up of cultured meat faces two major hurdles: the prohibitive cost of growth factors in serum‐free media and the accumulation of reactive oxygen species (ROS) in high‐density bioreactors. Recent advances in large‐scale production systems have emphasized the integration of automated monitoring and nutrient‐rich formulations to sustain the high‐density expansion of satellite cells while maintaining their myogenic potential (Kolkmann et al. 2022; Melzener et al. 2024). L‐carnosine, as a safe, natural, and low‐cost bioactive peptide, holds significant promise for the industrial‐scale production of cultured meat. Given that culture media – particularly recombinant growth factors – account for a substantial portion of production costs, identifying cost‐effective small molecules that can sustain cell expansion is a priority for the industry (Dai et al. 2024). Based on its known physiological functions, L‐carnosine could theoretically serve as a growth‐promoting supplement to alleviate the reliance on expensive mitogens. Furthermore, its established role as a potent antioxidant suggests it may help mitigate the oxidative stress typically encountered in high‐density bioreactor systems (Zhou et al. 2021), thereby enhancing bioprocess stability. However, the molecular mechanisms underlying these effects, particularly in the context of cultured meat production, remain underexplored. The molecular cascade regulatory network formed by MyoD, MyoG, and MyHC is the core mechanism of myogenic differentiation, and the involvement of different regulatory signals can influence the type and function of muscle fibers. However, there is currently a lack of systematic research on whether L‐carnosine can influence this myogenic differentiation cascade, particularly its potential selective regulatory role in the formation of fast‐twitch and slow‐twitch muscle fibers (Braun and Gautel 2011; Vicente‐García et al. 2022; Zammit 2017). Additionally, oxidative stress is considered an important factor influencing satellite cell activity and myogenic capacity, with excessive accumulation of ROS inhibiting proliferation and disrupting the differentiation process (Kozakowska et al. 2015; Lian et al. 2022; Summer et al. 2019). But its specific mechanism in regulating ROS homeostasis in bovine satellite cells remains unclear.

Existing evidence indicates that the growth, differentiation, and protein synthesis of muscle cells are precisely regulated by multiple signaling pathways (Vainshtein and Sandri 2020). However, during the action of L‐carnosine, the key molecular networks and signaling pathways involved remain poorly understood due to the lack of systematic mechanistic studies, which limits a deeper understanding of its mechanism and impacts its potential applications in beef cattle production and cultured meat technology. Yanbian cattle, one of the top five indigenous cattle breeds in China, are renowned for their unique flavor, tenderness, and high marbling scores. In the context of cultured meat, selecting seed cells from high‐quality breeds like Yanbian cattle is crucial for producing premium meat products that retain breed‐specific sensory characteristics, offering a competitive advantage over generic cultured beef (Li 2023). Based on this, the study uses Yanbian cattle skeletal muscle satellite cells as a model, establishes an in vitro culture system, and compares the effects of different concentrations of L‐carnosine on satellite cell proliferation, myogenic differentiation, and antioxidant capacity, combined with transcriptome sequencing and functional validation experiments, aiming to elucidate the molecular basis of its regulatory effects and identify key signaling networks that may be involved. This study will provide a theoretical basis for revealing the biological role of L‐carnosine in the development of bovine skeletal muscle, as well as offer new insights and experimental support for improving beef quality and advancing cultured meat technology based on endogenous bioactive peptides.

## 2 Materials and Methods

### 2.1 Materials

The DMEM high‐glucose medium, fetal bovine serum (FBS), 0.25% trypsin, Earle's Balanced Salt Solution (EBSS), and horse serum (HS) were sourced from Gibco (USA). TRIZOL Reagent and the BCA protein assay kit were acquired from Thermo Fisher Scientific (USA). Calcium/magnesium‐free phosphate buffer, penicillin–streptomycin solution, 0.4% Trypan Blue staining solution, Live/Dead cell fluorescent staining kit, Rainbow Marker, glycine, RIPA lysis buffer, protease/phosphatase inhibitor cocktail, RNase A, L‐carnosine, and anti‐fluorescence quenching mounting medium (with DAPI) were provided by Solarbio (China). SuperReal PreMix Plus and the FastKing RT Kit (with gDNase) were sourced from TIANGEN Biotech (China). Primary antibodies against housekeeping proteins, Pax7, PDGFRA (rabbit and mouse), MyoD, MyoG, CDK1, CDK2, and P21 were obtained from BIOSS (China). 4% paraformaldehyde, primers, and Triton X‐100 were supplied by Sangon Biotech (China). Bovine serum albumin (BSA) and dimethyl sulfoxide (DMSO) were procured from Sigma‐Aldrich (USA). FITC‐conjugated goat anti‐rabbit/anti‐mouse secondary antibodies were acquired from EarthOx (USA). Absolute ethanol and methanol were supplied by Kermel (China). 20× TBST buffer, 10× electrophoresis transfer buffer, rapid blocking solution, SDS‐PAGE electrophoresis buffer (10×), ROS assay kit, MDA assay kit, GSH‐Px assay kit, and SOD assay kit were obtained from Beyotime Biotechnology (China). ECL chemiluminescence kit and 5 × loading buffer were sourced from Yeasen Biotechnology (China). The 12.5% SDS‐PAGE gel preparation kit was supplied by EpiZyme Biotechnology (China). The CCK‐8 assay kit was obtained from APExBIO (USA). The PVDF membrane was acquired from Millipore (USA). The EdU assay kit was provided by RiboBio (China). The Myosin Heavy Chain (MyHC) primary antibody was supplied by Proteintech (USA).

### 2.2 Cell Isolation, Culture, and Identification

Fresh semitendinosus muscle tissue from Yanbian cattle was obtained, and the fascia and fat were removed under sterile conditions. To ensure biological independence, muscle samples from the three healthy Yanbian cattle (*n* = 3 biological replicates) were processed and digested independently. Primary cells were isolated using Pronase digestion and purified through the differential adhesion method. To purify satellite cells, the cell suspension was subjected to differential adhesion for 2 h at 37°C. The supernatant (containing unattached satellite cells) was collected and transferred to new collagen‐coated plates, leaving fibroblasts attached to the original dishes. These three independent cell populations were maintained as separate biological replicates throughout all subsequent experiments. The bovine tissue samples used in this study were obtained from a certified commercial slaughterhouse. All sample collection procedures complied with relevant animal welfare guidelines. The experimental protocol was reviewed and approved by the Institutional Animal Care and Use Committee (IACUC) of Yanbian University (Approval No. YD20240510060).

The cells were cultured in DMEM high‐glucose medium supplemented with 10% fetal bovine serum (FBS). Immunofluorescence was used to detect the expression of *Pax7*, MyoD, and Desmin proteins, while RT‐qPCR was employed to measure the expression levels of myogenic marker genes (MyoD, MyoG, MyHC) after differentiation induction to assess cell purity. Differentiation was induced by substituting FBS with 2% horse serum.

### 2.3 Study on the Mechanism of L‐Carnosine and Its Components on Skeletal Muscle Satellite Cell Proliferation

#### 2.3.1 Effects of L‐Carnosine on Proliferation and Toxicity of Skeletal Muscle Satellite Cells

Cell proliferation activity was assessed: When the density of skeletal muscle satellite cells reached over 80%, they were seeded into a 96‐well plate at a density of 3000 cells/well and treated with different concentrations of L‐carnosine (2.5, 5, 10, 20, 40 mM). The selection of L‐carnosine concentrations (2.5–40 mM) was primarily determined by our preliminary dose–response screening, which evaluated cell viability and proliferation to identify the optimal non‐toxic therapeutic window. This range also aligns with the typical physiological concentrations found in bovine skeletal muscle. After 24, 48, 72, and 96 h of treatment, the 96‐well plate was removed, culture medium discarded, and cells were washed twice with PBS. Then, 10 μL of diluted CCK‐8 solution was added to each well, and the plate was incubated in a CO₂ incubator for 2–4 h. The absorbance at 450 nm was measured using a microplate reader, with six replicates for each treatment. The cell proliferation rate was assessed using the EdU (5‐ethynyl‐2′‐deoxyuridine) staining method. Cell toxicity was evaluated using the Calcein‐AM/PI double staining method.

#### 2.3.2 Effects of L‐Carnosine on the Expression of Proliferation‐Related Genes and Proteins in Skeletal Muscle Satellite Cells

RT‐qPCR was used to measure the expression of proliferation‐related genes. Total RNA was extracted using TRIZOL reagent and reverse transcribed into cDNA.

Gene expression analysis was conducted using the SYBR Green method on a real‐time fluorescent quantitative PCR instrument. GAPDH was used as the reference gene, and the relative gene expression was calculated using the 2^−ΔΔCT^ method. Primer sequences are listed in Table 1. Western blotting was used to detect the expression levels of proliferation‐related proteins *Pax7, CDK1, CDK2*, and *P21* in skeletal muscle satellite cells.

**TABLE 1:** Primer sequences.

Genes	Accessionnumber	Sequence (5′‐3′)	Length
*GAPDH*	NSO_4761240	ACTCTGGCAAAGTGGATGTTGTC GCATCACCCCACTTGATGTTG	95
*KI67*	XM_015460791.2	CAGTCAACACGCCGACCAGTAAG CATCACCTGCTGCTTCTCCTTCTG	144
*Pax7*	XM_015460690	TGCCCTCAGTGAGTTCGATT CGGGTTCTGACTCCACATCT	180
*CDK1*	NM_174016.2	CTCGGTGTCCTACTTCAAGTGTGTG TCGCAGACCTCCAGCATCCAG	87
*CDK2*	NM_001014934.1	GACGGAGCTTGTTATCGCAAATGC GAGGTACTGGCTTGGTCACATCTTG	108
*PCNA*	NM_001034494.1	GTCCAGGGCTCCATCTTGAAGAAAG GCTGCACCAAGGAGACATGAGAC	225
*P16*	NM 181024	TGCGAAGATCAGAGCGAAATACCC CAGTGATGTCGGATGGAACCAGATAC	80
*P53*	NM_205793.2	GCCCATCCTCACCATCATCACAC GCACAAACACGCACCTCAAAGC	225
*P21*	NM 176784	GTCCAGGGACGCGCATCAAATC CAAAGTCGAAGTTCCACCGCTCTC	69

### 2.4 Study on the Differentiation and Mechanism of L‐Carnosine on Skeletal Muscle Satellite Cells

#### 2.4.1 Induction of Differentiation of Yanbian Cattle Skeletal Muscle Satellite Cells

When the density of Yanbian cattle skeletal muscle satellite cells reaches 80%, the cells are transferred to a 6‐well plate and cultured in growth medium. When the cell density reaches 70%–80%, the growth medium is discarded, and the cells are washed twice with PBS. The pre‐prepared differentiation medium containing different concentrations (5, 10, and 20 mM) of L‐carnosine is then added for induction of differentiation.

#### 2.4.2 Effects of L‐Carnosine on the Expression of Differentiation‐Related Genes and Fiber Type in Yanbian Cattle Skeletal Muscle Satellite Cells

Total RNA was extracted using TRIZOL reagent and reverse transcribed into cDNA. Gene expression analysis was performed using the SYBR Green method on a real‐time fluorescence quantitative PCR instrument. GAPDH was used as the reference gene, and the relative gene expression was calculated using the 2^−ΔΔCT^ method. Primer sequences are listed in Table 2.

**TABLE 2:** Primer sequences.

Genes	Accession number	Sequence (5′‐3′)	Length
*GAPDH*	NSO_4761240	ACTCTGGCAAAGTGGATGTTGTC GCATCACCCCACTTGATGTTG	95
*Bax*	NM_176788	GCTTCAGGGTTTCATCCAGGATCG CAGACACTCGCTCAGCTTCTTGG	103
*Bcl2*	NM_173980.2	TGTGGATGACCGAGTACCTGAACC GCCAGACTGAGCAGTGCCTTC	87
*Caspase3*	NM_174314.2	GACAGTGGTGCTGAGGATGACATG TTCGCCAGGAAAAGTAACCAGGTG	96

#### 2.4.3 Western Blotting Analysis: Effect of L‐Carnosine on Differentiation‐Related Protein Expression in Yanbian Cattle Skeletal Muscle Satellite Cells

Total protein was extracted from the cells, quantified using the BCA method, and subjected to SDS‐PAGE electrophoresis followed by membrane transfer. The cells were incubated with specific primary antibodies (MyoD, MyoG, MyHC) and horseradish peroxidase‐conjugated secondary antibodies. Finally, the proteins were visualized using the ECL chemiluminescence method.

#### 2.4.4 Transcriptome Sequencing and Analysis

On the fourth day of differentiation, total RNA was extracted from the control group and the 10 mM L‐carnosine treatment group (*n* = 3 per group). RNA purity and concentration were assessed using a NanoDrop 2000 spectrophotometer (Thermo Fisher Scientific, USA), and RNA integrity was precisely evaluated using an Agilent 2100 Bioanalyzer (Agilent Technologies, USA). Eukaryotic mRNA was enriched using oligo (dT) magnetic beads and subsequently fragmented. The cDNA library was constructed, size‐selected using AMPure XP beads, and PCR‐amplified. Following library quality control using a Qubit 3.0 Fluorometer and Q‐PCR (effective concentration > 2 nM), high‐throughput sequencing was performed on the Illumina NovaSeq 6000 platform to generate 150 bp paired‐end (PE150) reads. For bioinformatics analysis, raw data were initially processed using fastp to remove adapter sequences and low‐quality reads (containing > 10% unknown nucleotides N or > 50% bases with Q‐score ≤ 10). The resulting high‐quality clean reads were aligned to the bovine reference genome (Bos_taurus.ARS‐UCD1.2) using HISAT (Kim et al. 2015). Transcripts were assembled using StringTie (Pertea et al. 2015), and gene expression levels were quantified as read counts using featureCounts and normalized to FPKM (Fragments Per Kilobase of transcript per Million mapped reads). Differential expression analysis between the two groups was executed using the DESeq2 R package. Differentially expressed genes (DEGs) were strictly defined using the thresholds of False Discovery Rate (FDR) < 0.05 and |log2FC| > 1. Finally, Gene Ontology (GO) and Kyoto Encyclopedia of Genes and Genomes (KEGG) functional enrichment analyses of the DEGs were performed using the clusterProfiler R package.

#### 2.4.5 Effect of L‐Carnosine on the Akt/mTOR/P70S6K Pathway in Yanbian Cattle Skeletal Muscle Satellite Cells

Total protein was extracted from the cells, quantified using the BCA method, and subjected to SDS‐PAGE electrophoresis followed by membrane transfer. The cells were incubated with specific primary antibodies (p‐Akt, Akt, p‐mTOR, mTOR, p‐P70S6K, P70S6K) and horseradish peroxidase‐conjugated secondary antibodies. Finally, the proteins were visualized using the ECL chemiluminescence method.

### 2.5 Antioxidant Effects of L‐Carnosine on Yanbian Cattle Skeletal Muscle Satellite Cells

Cell Grouping and Treatment: L‐Carnosine (10 mM), Astaxanthin (10 μM), NAC (2 mM). The drug concentrations were determined based on previous studies conducted by the research team (Naseem et al. 2025). After 24 h of treatment, oxidative stress was induced using H₂O₂. Cell viability was assessed by Calcein‐AM/PI double staining. ROS, SOD, GSH, and MDA assay kits were used to measure intracellular reactive oxygen species, superoxide dismutase, glutathione, and malondialdehyde levels, respectively. RT‐qPCR was used to detect the expression of apoptosis‐related genes *Bcl‐2*, *Bax*, and *Caspase‐3*. The sequence information is shown in Table 2.

### 2.6 Statistical Analysis

All experimental data were analyzed using SPSS 27.0 and GraphPad Prism 9.0 software. Data are presented as mean ± SD from at least three independent biological replicates (*n* = 3) and three technical replicates. Prior to analysis, data normality was verified using the Shapiro – Wilk test, and homogeneity of variance was assessed using Levene's test. For multi‐group comparisons, one‐way ANOVA was performed followed by Tukey's post hoc test. *p* < 0.05 was considered statistically significant.

## 3 Experimental Results

### 3.1 Successful Isolation and Identification of Bovine Skeletal Muscle Satellite Cells

**FIGURE 1:**
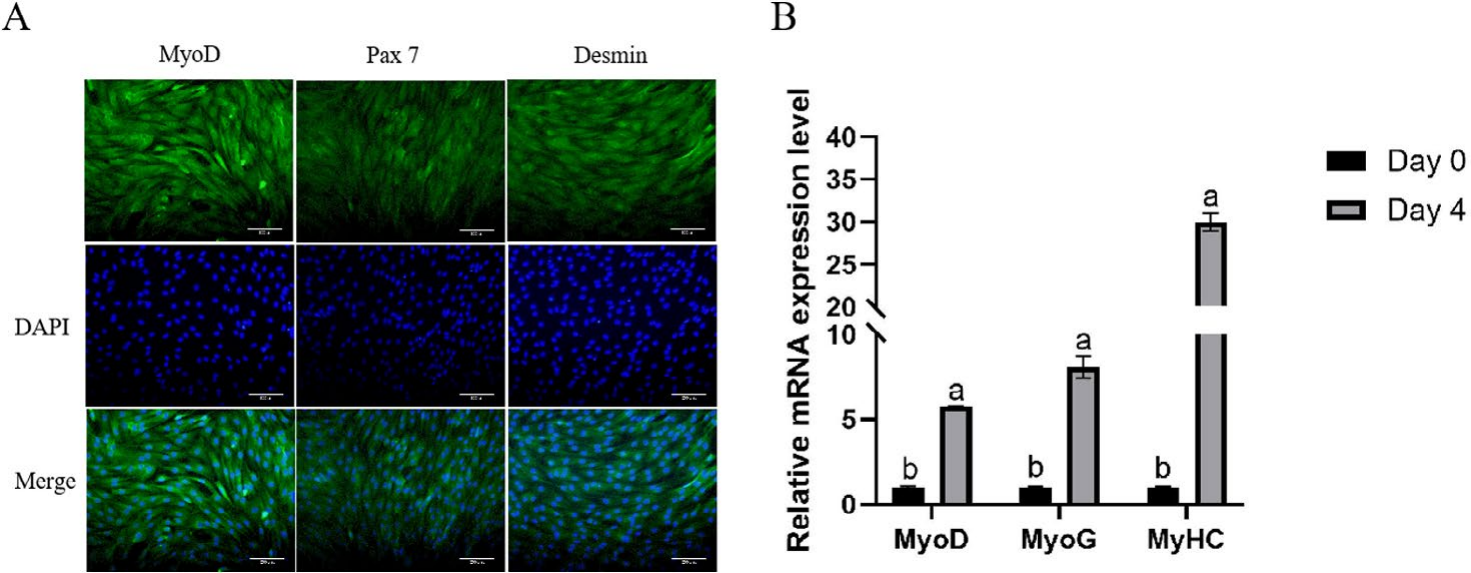
Identification and myogenic differentiation of Yanbian cattle skeletal muscle satellite cells (BSCs). (A) Immunofluorescence staining was performed to detect the expression of specific cellular markers: Pax7 (satellite cell marker), MyoD (myoblast proliferation marker), and Desmin (muscle‐specific structural protein), all shown in green. Nuclei were counterstained with DAPI (blue). Scale bar = 200 μm. (B) RT‐qPCR was used to measure the relative mRNA expression levels of myogenic marker genes (*MyoD*, *MyoG*, and *MyHC*). Cells were cultured either in growth medium (GM) or induced in differentiation medium (DM) for 4 days. Data are presented as the mean ± SD of independent biological replicates (*n* = 3). Statistical significance was determined using one‐way ANOVA. Different lowercase letters above the bars indicate highly significant differences (*p* < 0.001).

As shown in Figure 1A, the isolated cells were subjected to immunofluorescence staining, and the myogenic regulator MyoD, intermediate filament protein Desmin, and skeletal muscle satellite cell‐specific marker *Pax7* all exhibited positive signals (green). The cell nuclei were stained blue with DAPI (Figure 1A). The positive signals for all target proteins corresponded clearly with the nuclear location, confirming the specificity of the staining results and identifying the cells as skeletal muscle satellite cells. To assess the differentiation potential of the cells, samples were collected from growth medium (GM) culture (undifferentiated) and differentiation medium (DM) induced for 4 days. Total RNA was extracted using the Trizol method, reverse transcribed into cDNA, and mRNA expression levels of key myogenic differentiation markers (MyoD, MyoG, MyHC) were analyzed by RT‐qPCR (Figure 1B). The results showed that, compared to the GM group, the mRNA expression levels of MyoD, MyoG, and MyHC were significantly higher in the DM group after 4 days of induced differentiation (*p* < 0.001).

### 3.2 L‐Carnosine Promotes Skeletal Muscle Satellite Cell Proliferation With no Toxic Effects

**FIGURE 2:**
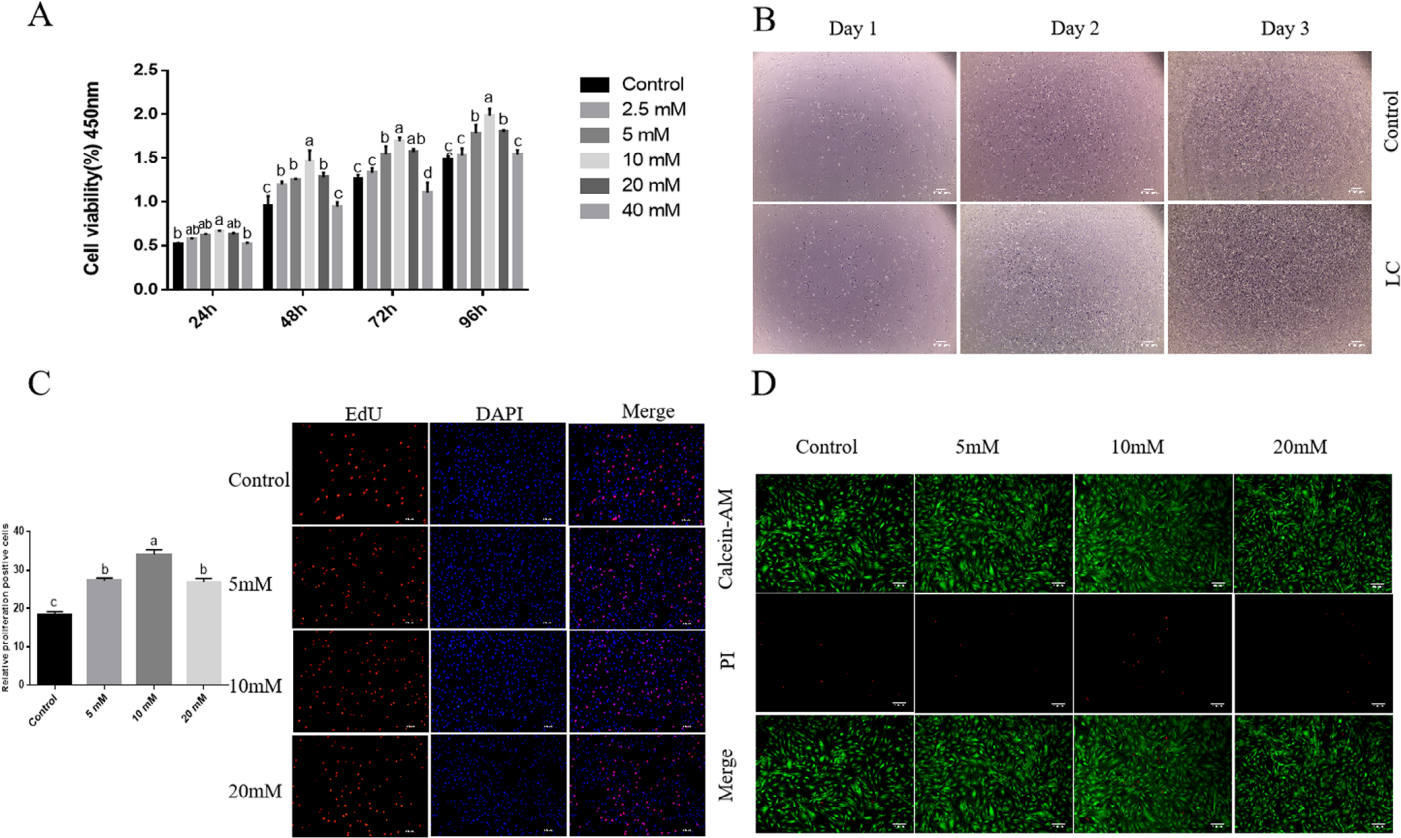
Effects of L‐carnosine on the viability and proliferation of Yanbian cattle BSCs. (A) Cell viability measured by CCK‐8 assay across 96 h (0–40 mM). (B) Representative bright‐field images of cell morphology (control vs. 10 mM); scale bar = 200 μm. (C) Proliferation quantified by EdU incorporation assay at 48 h (red: EdU; blue: DAPI); scale bar = 200 μm. (D) Cytotoxicity evaluation via Calcein‐AM/PI staining (green: Live cells; red: Dead cells); scale bar = 200 μm. Data represent mean ± SD from three independent biological replicates (*n* = 3). Different lowercase letters indicate statistical significance (*p* < 0.05, ne‐way or wo‐way ANOVA).

To determine the appropriate working concentration of L‐carnosine, the CCK‐8 assay was used to measure cell viability of Yanbian cattle skeletal muscle satellite cells (BSCs) treated with different concentrations (0, 2.5, 5, 10, 20, 40 mM) of L‐carnosine for 24, 48, 72, and 96 h (Figure 2A). The results showed that compared to the control group (0 mM), 5, 10, and 20 mM L‐carnosine significantly enhanced cell viability at 24, 48, 72, and 96 h (*p* < 0.05). However, treatment with 40 mM L‐carnosine for 72 and 96 h significantly decreased cell viability (*p* < 0.05). The 2.5 mM concentration had no significant effect on cell viability at 24, 72, and 96 h (*p* > 0.05). To further evaluate the cytotoxicity of the selected concentrations, the Calcein‐AM/PI double staining method was used to assess cell activity (Figure 2D). Compared to the control group, BSCs treated with 5, 10, and 20 mM L‐carnosine showed a significant increase in live cell density (green fluorescence) and a significant decrease in dead cell density (red fluorescence). This result confirmed that 5, 10, and 20 mM L‐carnosine had no significant cytotoxic effects on BSCs, so this concentration range was selected for subsequent experiments. To quantitatively evaluate the direct effect of L‐carnosine on cell proliferation, the EdU staining method was used to detect the DNA synthesis positivity rate of BSCs treated with different concentrations (5, 10, 20 mM) of L‐carnosine for 48 h (Figure 2C).

The results showed that compared to the control group (18.4% ± 0.8%), treatment with 5, 10, and 20 mM L‐carnosine significantly increased the EdU‐positive cell rate to 27.5% ± 1.2%, 34.1% ± 1.5%, and 26.8% ± 1.1%, respectively (*p* < 0.05; Figure 2C). Notably, the proliferative effect of 10 mM L‐carnosine was the most significant (*p* < 0.05 vs. all other groups), while no statistical difference was observed between the 5 and 20 mM groups (*p* > 0.05). Microscopic observations (Figure 2B) showed that after treatment with 10 mM L‐carnosine for 24, 48, and 72 h, BSCs exhibited normal morphology (early cells were predominantly fusiform or spindle‐shaped), firmly adhered to the surface, with no obvious vacuoles or granular material, and cell density increased with culture time.

### 3.3 L‐Carnosine Regulates Proliferation‐Related Gene and Protein Expression in Skeletal Muscle Satellite Cells

**FIGURE 3:**
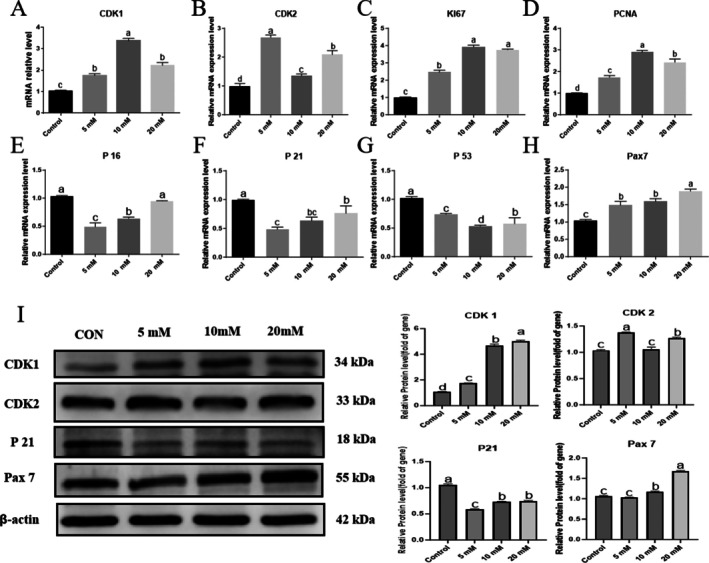
L‐Carnosine regulates the expression of proliferation‐related genes and proteins in Yanbian cattle BSCs. (A–D) RT‐qPCR was used to measure the mRNA levels of proliferation‐related genes. The expressions of *Ki67, PCNA*, and *CDK1* were highly upregulated (*p* < 0.001 vs. 0 mM), and *CDK2* was also significantly increased (*p* ≤ 0.010). (EG) RT‐qPCR was used to measure the mRNA levels of cell cycle inhibitors. *P21* and *P53* were significantly downregulated across all groups (*p* ≤ 0.021 and *p* ≤ 0.001, respectively). *P16* was significantly reduced at 5 and 10 mM (*p* < 0.001), but not at 20 mM (*p* = 0.092). *P21* and *P16* were lowest at 5 mM, and P53 was lowest at 10 mM. (H) RT‐qPCR was used to measure *Pax7* mRNA. Expression was significantly increased in all concentration groups (*p* < 0.001), with the 20 mM group showing the largest increase. (I) Western blot analysis was performed to examine the target protein expression (internal control: β‐Actin). Pax7 and CDK1 proteins were significantly upregulated in all groups (*p* < 0.05); CDK2 protein was upregulated in the 520 mM groups (*p* < 0.05), with no difference in the 10 mM group; P21 protein was significantly downregulated in all groups.

RT‐qPCR analysis showed (Figure 3A–H) that compared to the control group, treatment with 5, 10, and 20 mM L‐carnosine significantly upregulated the mRNA levels of proliferation‐related genes in Yanbian cattle skeletal muscle satellite cells (BSCs). Specifically, the expressions of *Ki67, PCNA*, and *CDK1* were highly elevated across all concentration groups (all *p* < 0.001). *CDK2* mRNA was also significantly increased (*p* < 0.001 for 5 and 20 mM; *p* = 0.010 for 10 mM), peaking at the 5 mM concentration, while *Ki67, PCNA*, and *CDK1* expressions were highest at 10 mM. Conversely, the mRNA expressions of cell cycle inhibitors were notably suppressed. *P21* was significantly downregulated across all treatments (*p* < 0.001, *p* = 0.002, and *p* = 0.021 for 5, 10, and 20 mM, respectively). *P16* expression was significantly reduced in the 5 and 10 mM groups (both *p* < 0.001), though it did not reach statistical significance at 20 mM (*p* = 0.092). *P53* mRNA was also significantly decreased (*p* = 0.001 for 5 mM; *p* < 0.001 for 10 and 20 mM), reaching its lowest level in the 10 mM group. At the same time, the mRNA level of the satellite cell marker *Pax7* was extremely significantly increased in all groups (all *p* < 0.001), with the 20 mM group showing the most prominent increase. Western blot results further confirmed (Figure 3I): Pax7 protein was significantly upregulated in all treatment groups (*p* < 0.05), CDK1 protein expression also increased synchronously (*p* < 0.05), while CDK2 protein was only significantly increased in the 5 and 20 mM groups (*p* < 0.05), with no difference between the 10 mM group and the control group (*p* > 0.05); In addition, P21 protein was significantly decreased in all concentration groups (*p* < 0.05).

### 3.4 L‐Carnosine Promotes Differentiation of Yanbian Cattle Skeletal Muscle Satellite Cells and Regulates Muscle Fiber Types

**FIGURE 4:**
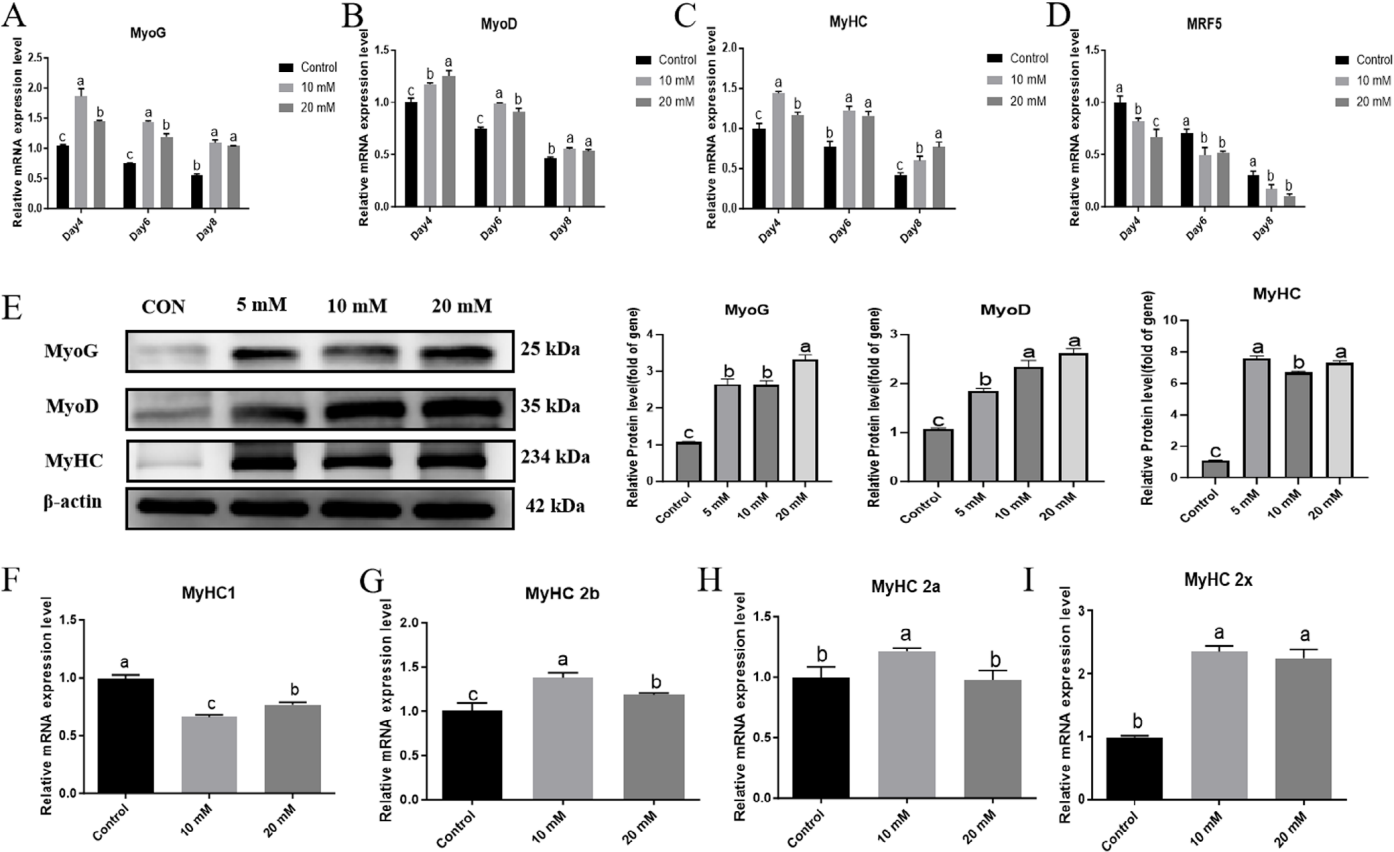
The effects of L‐carnosine on myogenic differentiation and muscle fiber types in Yanbian cattle BSCs. (A–D) RT‐qPCR analysis was used to measure the expression of differentiation marker genes. After treatment with 10/20 mM L‐carnosine for 4/6/8 days, MyHC, MyoD, and MyoG mRNA were significantly upregulated (*p* < 0.05 vs. CON), with peak expression at 4 days; *MRF5* mRNA was significantly downregulated (*p* < 0.05) and decreased with prolonged differentiation time. (E) Western blot analysis of protein expression after 96 h of differentiation (internal control: β‐Actin). The expression of MyoG, MyoD, and MyHC proteins was significantly increased in the 5, 10, and 20 mM groups (*p* < 0.05). (F–I) RT‐qPCR was used to measure myosin heavy chain isoforms. MyHC1 mRNA was downregulated in all groups (*p* < 0.05); MyHC2a mRNA was upregulated only in the 10 mM group (*p* < 0.05); *MyHC2b/MyHC2x* mRNA were upregulated in both the 10 and 20 mM groups (*p* < 0.05).

RT‐qPCR analysis showed (Figure 4A–E) that compared to the control group (0 mM), treatment with 10 and 20 mM L‐carnosine induced significant increases in the mRNA expression levels of muscle‐specific differentiation marker genes (MyoG, MyoD, and MyHC) in Yanbian cattle skeletal muscle satellite cells across 4, 6, and 8 days of differentiation. Overall, the expressions of these myogenic markers peaked at day 4, followed by day 6, and were lowest at day 8. Specifically for MyoG, both 10 and 20 mM treatments significantly upregulated its expression compared to the control at day 4 and day 6 (all *p* < 0.001). Furthermore, the 10 mM group showed significantly higher expression than the 20 mM group at both of these early time points (both *p* < 0.001). By day 8, both L‐carnosine treatments remained significantly higher than the control (*p* < 0.001), but the difference between 10 and 20 mM was no longer statistically significant (*p* = 0.960). For MyoD, its expression was also strongly induced by L‐carnosine. At day 4, both treatments significantly increased MyoD levels compared to the control (*p* < 0.001), with the 20 mM group showing a slightly higher expression than the 10 mM group (*p* = 0.044). At day 6, both treatments remained highly upregulated against the control (*p* < 0.001), though the difference between the two doses became marginal (*p* = 0.057). By day 8, the 10 mM group maintained a significantly higher expression than the control (*p* = 0.019), while there was no statistical difference between the 10 and 20 mM doses (*p* = 0.998).

Similarly, MyHC expression was significantly elevated. At day 4, both concentrations significantly increased MyHC levels compared to the control (e.g., *p* = 0.022 for 20 mM vs. control), with 10 mM showing the strongest induction (*p* < 0.001 vs. 20 mM). At day 6, both treatments were highly upregulated against the control (*p* < 0.001), with no statistical difference between the two doses (*p* = 0.779). By day 8, the 20 mM group maintained the highest MyHC expression (*p* < 0.001 vs. control; *p* = 0.026 vs. 10 mM).

In contrast, the mRNA expression of the muscle differentiation inhibitor *MRF5* was significantly downregulated by the treatments. At day 4, both 10 and 20 mM treatments significantly reduced *MRF5* levels compared to the control (*p* = 0.011 and *p* < 0.001, respectively). This significant suppression was maintained at day 6 (*p* = 0.002 for 10 mM; *p* = 0.010 for 20 mM). Overall, *MRF5* expression exhibited a continuous decreasing trend with prolonged differentiation time.

Western blot results further confirmed (Figure 4E): after 96 h of differentiation with 5, 10, and 20 mM L‐carnosine treatment, the expression of MyoG, MyoD, and MyHC proteins was significantly upregulated compared to the control group (*p* < 0.05).

Muscle fiber type‐specific analysis showed (Figure 4F–I) that L‐carnosine treatment significantly altered the expression of myosin heavy chain isoforms: MyHC1 mRNA was significantly reduced in all concentration groups (*p* < 0.05); MyHC2a mRNA was significantly increased only in the 10 mM group (*p* < 0.05), with no change in the 20 mM group; while MyHC2b and MyHC2x mRNA were significantly upregulated in both the 10 and 20 mM groups (*p* < 0.05).

### 3.5 Transcriptome Sequencing Identifies Signaling Pathways Involved in L‐Carnosine‐Induced Differentiation of Yanbian Cattle Skeletal Muscle Satellite Cells

**FIGURE 5:**
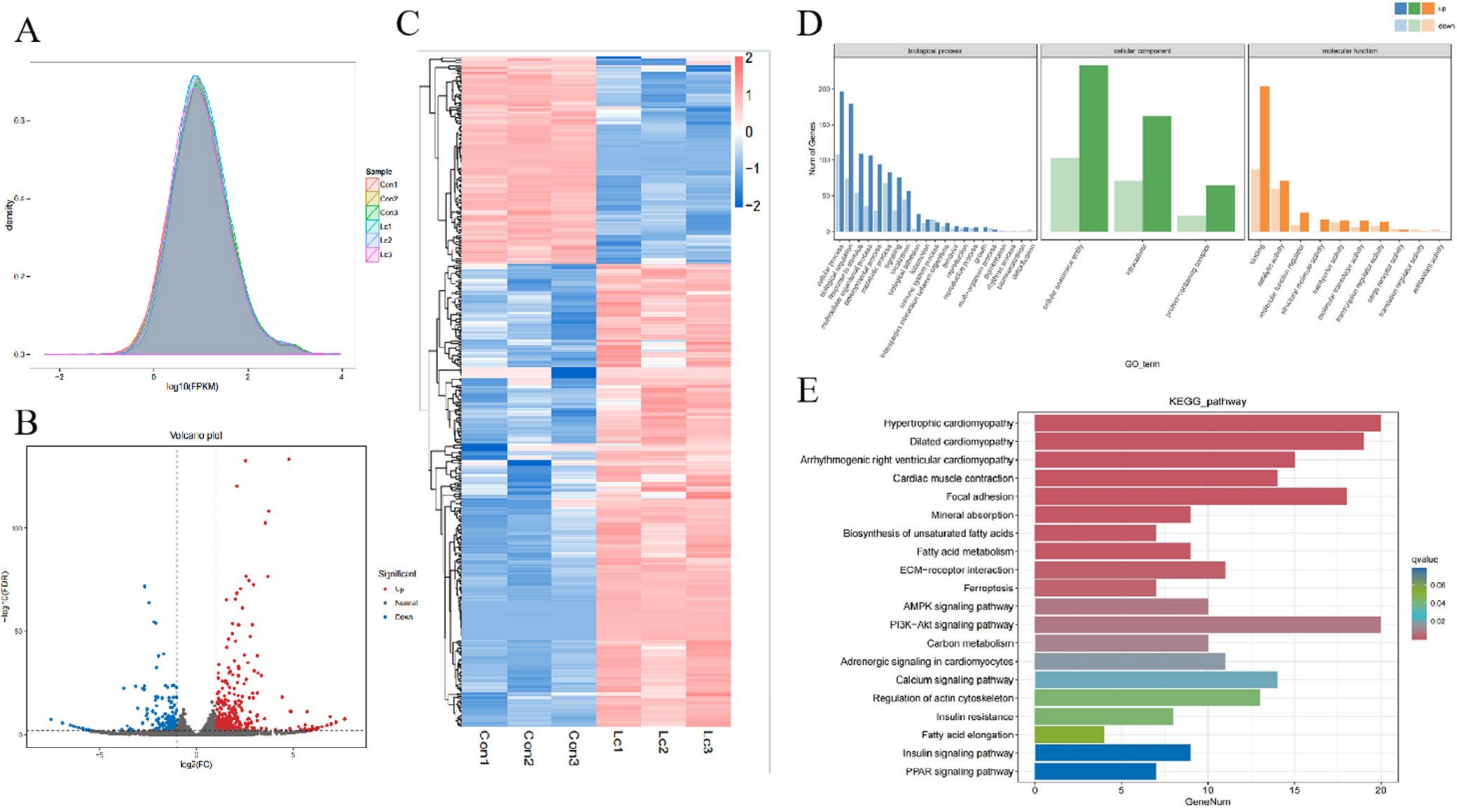
Transcriptomic profiling of Yanbian cattle skeletal muscle satellite cells (BSCs) following L‐carnosine treatment. (A) Schematic workflow for the identification and screening of differentially expressed genes (DEGs) via RNA sequencing. (B) Volcano plot illustrating the distribution of DEGs between the control (0 mM) and the 10 mM L‐carnosine‐treated groups. The screening thresholds were strictly set to false discovery rate (FDR) < 0.01 and fold change ≥ 2. A total of 449 DEGs were identified, including 310 significantly upregulated genes (red dots) and 139 significantly downregulated genes (blue dots). (C) Hierarchical clustering heatmap of the 449 DEGs. The horizontal axis represents the individual biological replicates (*n* = 3 per group), and the vertical axis represents the individual DEGs. The color gradient indicates low (dark blue) to high (red) relative gene expression levels. (D) Gene ontology (GO) functional enrichment analysis of the DEGs. The top enriched terms are categorized into biological processes (BP; e.g., developmental regulation, tissue morphogenesis, and metabolic homeostasis), cellular components (CC; e.g., cytoskeleton), and molecular functions (MF; e.g., enzyme activity and transcription regulation). (E) Kyoto encyclopedia of genes and genomes (KEGG) pathway enrichment scatter plot. Core enriched pathways include the PI3K‐AKT signaling pathway (showing the highest enrichment level), cardiomyopathy‐related pathways, AMPK/PPAR metabolic pathways, and calcium signaling.

Differential gene analysis based on RNA‐seq data (Figure 5A,B) showed that in Yanbian cattle skeletal muscle satellite cells (BSCs) treated with 10 mM L‐carnosine, 449 differentially expressed genes (DEGs) were identified using False Discovery Rate (FDR) < 0.01 and Fold Change ≥ 2 as thresholds, with 310 significantly upregulated and 139 significantly downregulated (Figure 5B). Among the 310 upregulated genes, a prominent cluster of muscle structural and functional genes was identified. Specifically, markers critical for skeletal muscle development and fast‐twitch fiber specification – such as MYH8, MYL1, ACTA1, and the fast‐twitch calcium ATPase ATP2A1 – were extremely significantly enriched, firmly corroborating our in vitro qPCR and Western blot results. Conversely, the downregulated group primarily included genes associated with cellular stress responses and tissue remodeling (such as HSPA9, TXNRD1, and TIMP3), reflecting a shift toward a robust, stable differentiated state. To provide more comprehensive data, the top 10 significantly upregulated and downregulated genes are detailed in Table S1.

Cluster heatmap analysis (Figure 5C) revealed that the expression profile of the DEGs exhibited distinct grouping characteristics, with significant clustering of gene expression patterns between samples (horizontal axis: experimental samples; vertical axis: DEGs; color scale: dark blue for low expression to red for high expression), suggesting that functionally related genes may participate in coordinated regulation.

GO functional annotation (Figure 5D) revealed that these DEGs were primarily enriched in three major categories: biological processes (regulation of multicellular organism development, tissue morphogenesis, maintenance of metabolic homeostasis, cell signaling, and host‐pathogen interaction), cellular components (cytoskeletal structure, etc.), and molecular functions (hydrolase/oxidoreductase activity, transcription factor binding, protein modification regulation, and transmembrane transport).

KEGG pathway enrichment analysis (Figure 5E) further indicated that the DEGs were significantly involved in cardiac pathological processes (hypertrophic/dilated cardiomyopathy pathways), energy metabolism reprogramming (AMPK/PPAR signaling pathways, fatty acid metabolism), and cell signaling transduction (PI3K‐AKT and calcium signaling pathways), with the PI3K‐AKT pathway showing the highest enrichment level (Figure 5E), suggesting that it plays a central role in the differentiation of Yanbian cattle skeletal muscle satellite cells by regulating cell proliferation, differentiation, and ion balance.

### 3.6 L‐Carnosine Activates the Akt/mTOR/P70S6K Signaling Pathway to Promote Skeletal Muscle Satellite Cell Differentiation

**FIGURE 6:**
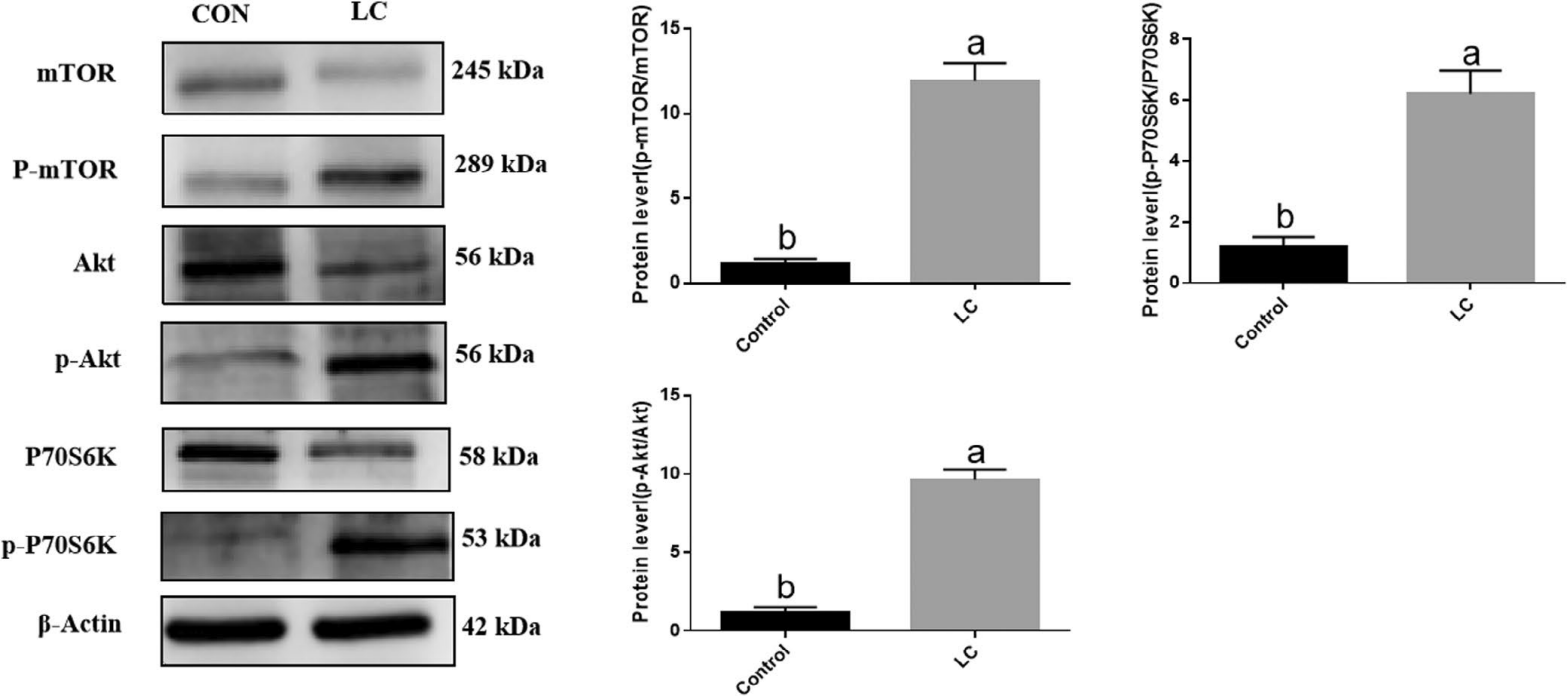
L‐carnosine activates the Akt/mTOR/P70S6K signaling pathway in Yanbian cattle skeletal muscle satellite cells (BSCs). > Western blot analysis was performed to evaluate the expression levels of key mechanistical proteins following L‐carnosine treatment. Representative immunoblots and corresponding densitometric quantification demonstrate that, compared to the control group (CON, 0 mM), L‐carnosine treatment significantly increased the relative phosphorylation levels of Akt (p‐Akt), mTOR (p‐mTOR), and P70S6K (p‐P70S6K) (*p* < 0.05). β‐Actin was utilized as the internal loading control. Data are presented as the mean ± SD of independent biological replicates (*n* = 3). Statistical significance was determined using one‐way ANOVA followed by Tukey's post hoc test. Different lowercase letters above the bars indicate statistically significant differences between treatment groups.

Western blotting analysis (Figure 6) showed that, compared to the control group (CON), L‐Carnosine treatment significantly upregulated the phosphorylation levels of key nodes in the Akt/mTOR/P70S6K signaling pathway in Yanbian cattle skeletal muscle satellite cells: p‐Akt, p‐mTOR, and p‐P70S6K protein expression levels were all significantly increased (*p* < 0.05), suggesting that L‐Carnosine may regulate the cell differentiation process by activating the Akt/mTOR/P70S6K signaling pathway.

### 3.7 L‐Carnosine Maintains Skeletal Muscle Satellite Cell Differentiation Through Antioxidant and Anti‐Apoptotic Effects

**FIGURE 7:**
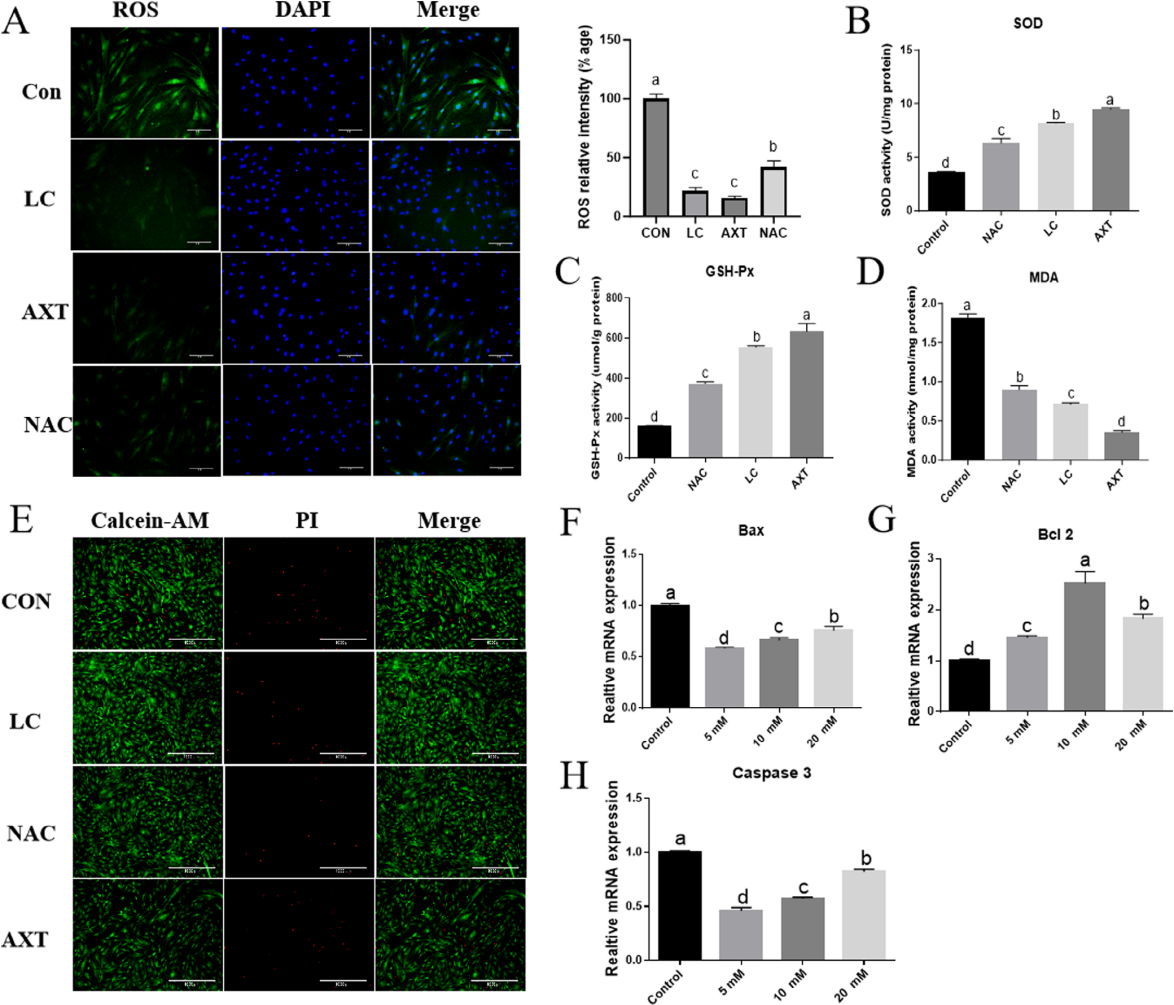
Protective effects of L‐carnosine (LC) against H_2_O_2_‐induced oxidative stress and apoptosis in BSCs. (A) ROS fluorescence levels following induction with 600 μM H_2_O_2_ (2 h) and treatment with LC (10 mM), NAC (2 mM), or AST (10 μM); scale bar = 200 μm. (B–D) SOD activity, GSH content, and MDA levels in treated cells. (E) Calcein‐AM/PI staining for cell viability (green: Live; red: Dead); scale bar = 200 μm. (F–H) RT‐qPCR analysis of apoptosis‐related genes (Bax, Bcl‐2, and Caspase 3). All data represent the mean ± SD of three independent biological experiments (*n* = 3 cattle). Statistical significance was determined using one‐way ANOVA followed by Tukey's test; different lowercase letters indicate *p* < 0.05.

Calcein‐AM/PI dual staining results (Figure 7E) showed that, compared to the control group, the BSCs treated with 10 mM L‐Carnosine (LC), 2 mM N‐acetylcysteine (NAC), and 10 μM Astaxanthin (AST) exhibited significantly increased live cell density (green) and significantly decreased dead cell density (red), confirming that none of these treatments exhibited cytotoxicity and thus were selected for subsequent experiments. Oxidative stress analysis (Figure 7A) indicated that, after 2 h of induction with 600 μM H₂O₂, the levels of reactive oxygen species (ROS) in the LC, NAC, and AST treatment groups were significantly lower than those in the control group (*p* < 0.05), with the order AST < LC < NAC. Antioxidant enzyme activity assays further demonstrated (Figure 7B–D) that the activities of superoxide dismutase (SOD) and the content of glutathione peroxidase (GSH) were significantly increased in all three groups (*p* < 0.05), with a gradient effect of AST > LC > NAC. At the same time, the lipid oxidation damage marker malondialdehyde (MDA) levels significantly decreased (*p* < 0.05), with a gradient of AST < LC < NAC (Figure 7F–H). Additionally, L‐Carnosine concentration gradient experiments (5/10/20 mM) confirmed (figure not shown) that as the concentration increased, the expression of pro‐apoptotic gene Bax and apoptotic execution gene Caspase 3 mRNA significantly decreased (*p* < 0.05), while the expression of the anti‐apoptotic gene Bcl‐2 mRNA significantly increased (*p* < 0.05).

## 4 Discussion and Conclusions

The results of this study show that L‐carnosine significantly promotes the in vitro proliferation of Yanbian cattle skeletal muscle satellite cells. The experiment shows that L‐carnosine treatment effectively increases cell viability, enhances the proportion of EdU‐positive cells (indicating active DNA synthesis), and confirms the increase in live cell numbers and decrease in dead cell numbers through Calcein‐AM/PI double staining, indicating that it promotes proliferation without cytotoxicity in the 5–20 mM concentration range. In terms of molecular mechanisms, L‐carnosine significantly downregulates the mRNA expression levels of cell cycle negative regulators *p53*, *p21*, and *p16*. This effect may stem from its powerful antioxidant capacity, which alleviates DNA oxidative damage and thereby reduces the activation of the *p53* pathway; at the same time, L‐carnosine may also indirectly inhibit *p21* expression by activating pro‐proliferation signaling pathways such as PI3K/Akt, or directly suppress *p16* transcription. On the other hand, L‐carnosine significantly upregulates the mRNA and protein expression levels of key cell cycle progression factors (CDK1, CDK2, PCNA, Ki67) and the transcription factor *Pax7*, which maintains stemness and proliferative potential. In summary, L‐carnosine may promote the in vitro proliferation of Yanbian cattle skeletal muscle satellite cells by clearing ROS, alleviating oxidative stress, inhibiting *p53/p21/p16*‐mediated cell cycle arrest, and synergistically activating proliferation‐related factors such as CDK1/CDK2 and maintaining stem cell characteristics regulated by Pax7, thus assisting the cells to pass through cell cycle checkpoints. The results of this study are consistent with the biological functions of L‐carnosine found in previous literature and expand the understanding of its mechanism of action. Consistent with reports by (Palin et al. 2020) in pig myoblasts, this study further confirms that L‐carnosine has significant antioxidant capacity, effectively alleviating cell oxidative damage, supporting its protective function in muscle precursor cells from various species. However, in Yanbian cattle satellite cells, L‐carnosine exhibits a more active pro‐proliferation phenotype under basal culture conditions. Its effect is not limited to the classic NEF2L2‐mediated antioxidant pathway but also reflects multi‐level regulation of the cell cycle progression. Specifically, L‐carnosine inhibits the p53‐p21‐p16 signaling axis, alleviating G1/S phase arrest, and at the same time, it synergistically enhances the expression of pro‐proliferative factors such as CDK1, CDK2, PCNA, Ki67, and Pax7, from mRNA to protein levels, thereby driving cell cycle progression. This result is highly consistent with the activation of the mTOR pathway under basal conditions reported in pig cells, suggesting that the upstream regulation of the mTOR signaling pathway may be a common mechanism of the pro‐proliferative effect of L‐carnosine across species. It is worth noting that the action pattern of L‐carnosine shows significant condition dependence: under oxidative stress conditions, it primarily exerts cell protective effects through pathways such as NEF2L2 and p38 MAPK; while under physiological or low‐stress conditions, it tends to drive cell proliferation and stemness maintenance via pro‐growth signaling pathways such as mTOR.

In addition to promoting proliferation, this study systematically reports the regulatory effects of L‐carnosine on myogenic differentiation of satellite cells in Yanbian cattle skeletal muscle and its potential mechanisms. The addition of L‐carnosine (especially 10 mM) for inducing differentiation significantly increases the formation rate and fusion rate of multinucleated myotubes. Molecular analysis shows that at the early stage of differentiation (day 4), the gene and protein expression of key myogenic regulators MyoD, MyoG, and the contractile protein MyHC are significantly upregulated, indicating that L‐carnosine effectively initiates the myogenic differentiation program. Notably, as the differentiation progresses (days 6–8), the expression levels of these markers show a downward trend, which is consistent with the typical characteristics of terminal muscle cell differentiation – transitioning from the active biosynthesis phase (high expression of myogenic transcription factors and structural proteins) to the functional maintenance phase (formation of mature myotubes). Further mechanistic studies found that L‐carnosine treatment significantly activates the Akt/mTOR/P70S6K signaling pathway in Yanbian cattle skeletal muscle satellite cells. This pathway is a central hub for regulating protein synthesis and cell growth, playing a key role in myogenesis (Ge et al. 2024; Hu et al. 2010). We speculate that L‐carnosine may directly or indirectly (e.g., by mimicking growth factors or binding specific receptors such as IGF1R) trigger the upstream PI3K/Akt signaling, thereby activating mTORC1 and its downstream effector molecule P70S6K, ultimately driving myogenic gene expression and myotube formation. Interestingly, this study also observed the regulatory effects of L‐carnosine on muscle fiber type composition: it selectively upregulates the mRNA expression of fast‐twitch fiber markers (MyHC 2a, 2 ×, 2b) while inhibiting the expression of slow‐twitch fiber markers (MyHC 1). This shift toward a fast‐twitch fiber phenotype may be related to the preferential upregulation of fast‐twitch fiber‐related transcription factors (e.g., MyoD) by the PI3K‐Akt–mTOR pathway activated by L‐carnosine, the inhibition of slow‐twitch fiber regulatory factors, as well as its ability to buffer the glycolytic acidic microenvironment and enhance glycolytic enzyme activity, thus facilitating energy supply and phenotype maintenance in fast‐twitch fibers that rely on anaerobic metabolism. Compared with the study by (Kalbe et al. 2023) on pig myoblasts, both studies indicate that carnosine plays an important biological role in muscle precursor cells across different species, but its specific effects and molecular mechanisms show clear condition dependence and process specificity. Kalbe et al. found that under oxidative stress conditions, carnosine primarily enhances cellular protection by activating the NEF2L2 antioxidant pathway and inhibiting the p38 MAPK signaling; while under basal conditions, it may promote proliferation through the mTOR pathway. This study further extends this understanding, finding that under differentiation‐inducing conditions, L‐carnosine significantly promotes myotube formation and the expression of myogenic markers (MyoD, MyoG, MyHC) by activating the Akt/mTOR/P70S6K signaling axis that regulates protein synthesis and myogenesis, indicating that mTOR signaling plays a crucial role in both proliferation and differentiation mediated by carnosine. Moreover, this study reports for the first time that L‐carnosine can regulate muscle fiber type composition, preferentially promoting the expression of fast‐twitch fiber‐related genes. This phenomenon may be related to its preferential activation of the PI3K‐Akt–mTOR pathway, its buffering effect on the glycolytic microenvironment, and energy metabolism reprogramming, which also provides a potential functional link to explain the higher content of carnosine in glycolytic‐type muscles. A critical novelty of this study is the specific upregulation of fast‐twitch muscle fiber markers (MyHC2a, MyHC2b, MyHC2x) driven by L‐carnosine. The mechanistic link between this phenotypic shift and the Akt/mTOR/P70S6K pathway warrants deeper interpretation. mTORC1 is not merely a central hub for protein synthesis; it is a master regulator of metabolic reprogramming. Fast‐twitch (Type II) fibers are highly reliant on glycolytic metabolism and demand robust protein synthesis for hypertrophy. Activation of the Akt/mTOR axis inherently favors a glycolytic metabolic state (a Warburg‐like effect in normal cells), which metabolically supports the differentiation and maintenance of Type II fibers over the oxidative Type I fibers (which are more dependent on AMPK/PGC‐1α signaling). Therefore, L‐carnosine actively drives fast fiber differentiation through a dual mechanism: biochemically via mTOR‐induced protein synthesis, and metabolically by buffering the consequent glycolytic acidosis. To fully situate the biological significance of L‐carnosine in muscle development, it is essential to compare it with other abundant bioactive peptides, such as creatine and taurine. While taurine primarily acts as an osmolyte and calcium regulator, and creatine serves as a rapid spatial energy buffer (via phosphocreatine), L‐carnosine is uniquely characterized by the pKa (6.83) of its imidazole ring, which confers exceptional intracellular pH‐buffering capacity. This specific trait provides a vital physiological advantage for fast‐twitch glycolytic fibers, which generate substantial lactic acid during metabolism. In conclusion, the effects of carnosine are clearly dependent on the environmental and cellular state: under stress conditions, its main role is cellular protection; during the proliferation phase, it mainly promotes mitosis; and during the differentiation phase, its core function is to drive myogenesis and fiber type specialization. Its multiple biological functions collectively support its key position in muscle development and metabolic adaptation.

The protective effects of L‐carnosine on Yanbian cattle skeletal muscle satellite cells are clearly reflected in its significant antioxidant and anti‐apoptotic abilities. Experimental evidence confirms that L‐carnosine treatment robustly reduces intracellular reactive oxygen species (ROS) and the lipid peroxidation product malondialdehyde (MDA), while simultaneously increasing the activity of key antioxidant enzymes like superoxide dismutase (SOD) and enhancing glutathione (GSH‐Px) content. It is crucial to delineate that this potent antioxidant capacity is multifaceted and mediated through a dual‐action mechanism. Directly, the imidazole ring of L‐carnosine functions as a chemical scavenger of free radicals and a chelator of transition metals (such as iron and copper), thereby preventing the highly reactive Fenton reaction and stabilizing cell membrane and mitochondrial functions. Indirectly, L‐carnosine exerts its cytoprotective effects via signal transduction; the activation of the PI3K/Akt pathway can subsequently promote the nuclear translocation of antioxidant response elements (e.g., Nrf2) to upregulate the endogenous antioxidant defense system. Furthermore, this pathway actively suppresses pro‐apoptotic signaling, as evidenced by our observed downregulation of p53 and Bax. This synergistic direct and indirect mechanism ensures that satellite cells are robustly protected from oxidative damage and apoptosis during the intense metabolic shifts of proliferation and differentiation. This powerful antioxidant capacity is directly linked to its anti‐apoptotic effects: L‐carnosine treatment significantly reduces the mRNA expression of pro‐apoptotic gene Bax and apoptosis execution gene Caspase 3, while increasing the expression of anti‐apoptotic gene Bcl‐2. This suggests that L‐carnosine can alleviate oxidative stress damage, block the activation of the mitochondrial apoptotic pathway (inhibiting Bax translocation, maintaining mitochondrial membrane potential), and possibly synergistically activate pro‐survival signaling pathways such as PI3K/Akt, ultimately inhibiting the Caspase cascade and maintaining cell survival. In line with this, Rahman et al. (Rahman et al. 2024) similarly reported in a Dex‐induced C2C12 myotube atrophy model that carnosine treatment significantly reduces intracellular ROS levels, inhibits FoxO3a transcriptional activity, and downregulates the expression of atrophy‐related E3 ubiquitin ligases (e.g., Atrogin‐1, MuRF‐1), thereby effectively delaying muscle atrophy progression. Particularly noteworthy is that the study emphasizes the necessity of the overall molecular structure of carnosine for its function, as its hydrolyzed components – β‐alanine and histidine mixture (HA) failed to replicate the equivalent protective effects. This result is highly consistent with the central role of L‐carnosine as an intact molecule in regulating cell fate in this study, suggesting that its biological functions depend on the integrity of the dipeptide structure and potentially related specific spatial conformations and receptor interaction patterns. A comparison of the two studies from a mechanistic perspective reveals that carnosine utilizes antioxidant as its core mechanism under different physiological and pathological conditions (atrophy stress and normal myogenesis). However, the downstream signaling pathways and effector factors exhibit model specificity: in the stress model, it primarily prevents protein degradation by inhibiting the FoxO3a–ubiquitin‐proteasome system, whereas in the differentiation model, it promotes protein synthesis and myotube maturation via the Akt/mTOR axis. This contextual dependency in the selection of signaling pathways highlights the multifunctionality and adaptability of carnosine as an endogenous active molecule in maintaining cellular homeostasis. In summary of the findings of this study, L‐carnosine optimizes the in vitro culture performance of Yanbian cattle skeletal muscle satellite cells through multiple pathways, including promoting proliferation, inducing differentiation (toward fast‐twitch muscle fibers), enhancing antioxidant defense, and inhibiting apoptosis. These effects are primarily attributed to its activation of the Akt/mTOR/P70S6K signaling axis and its inherent potent antioxidant properties. From an application perspective, as a dipeptide naturally found in meat, L‐carnosine holds great potential as a safe and efficient culture medium additive for cultured meat production: it can directly promote myotube formation and maturation, improving the texture of cultured meat (particularly simulating fast‐twitch muscle fiber characteristics); its antioxidant properties help maintain cell viability under high‐density culture conditions, enhancing production efficiency; and it avoids regulatory concerns associated with exogenous growth factors. Furthermore, this study provides important molecular mechanistic insights for understanding how L‐carnosine improves livestock meat quality (e.g., by affecting muscle fiber types) and for developing functional feed additives that combat muscle atrophy. While our in vitro findings provide compelling evidence for L‐carnosine's efficacy in cellular models (relevant to cultured meat production), translating these benefits to traditional livestock farming necessitates future in vivo dietary supplementation trials. Ruminants pose a unique physiological challenge due to the potential degradation of oral peptides by rumen microbes. Therefore, future studies must focus on utilizing rumen‐protected L‐carnosine or supplementing its rate‐limiting precursors (such as β‐alanine and histidine) in the diet of live Yanbian cattle. Evaluating how dietary supplementation impacts in vivo muscle hypertrophy, feed conversion efficiency, and terminal meat quality traits (such as marbling, tenderness, and color stability) will be the critical next step to validate L‐carnosine as a functional feed additive in the beef industry.

In conclusion, this study demonstrates that L‐carnosine promotes myogenic differentiation and fast‐twitch fiber specification in bovine satellite cells via the Akt/mTOR/P70S6K axis and potent antioxidant mechanisms. These findings offer promising biological solutions for the cultured meat industry: L‐carnosine's antioxidant properties can mitigate oxidative damage during large‐scale cell expansion, while its promotion of fast‐twitch fibers can improve the texture and organoleptic profile of cultured beef.

## Author Contributions

**Huaina Jin:** conceptualization, methodology, software, data curation, writing – original draft. **Xuanying Xin:** conceptualization, methodology, formal analysis, writing – original draft, investigation. **Sajida Naseem:** conceptualization, visualization, investigation. **Xiangzi Li:** writing – review and editing, visualization, project administration, resources, funding acquisition. **Bin Sun:** conceptualization, methodology, software, investigation, writing – original draft. **Sungkwon Park:** funding acquisition, visualization, project administration, resources, writing – review and editing. **Seong‐Ho Choi:** investigation, conceptualization, validation.

## Funding

This study was supported by the Natural Science Foundation of Jilin Province (Grant No. YDZJ202101ZYTS105), the National Natural Science Foundation of China (funding number: 32060767) and the Research Fund of Engineering Research Center of North‐East Cold Region Beef Cattle Science & Technology Innovation, Ministry of Education, and the “111” Project (D20034), China.

## Conflicts of Interest

The authors declare no conflicts of interest.

## Supporting information


**Table S1:** Top 10 significantly upregulated and downregulated differentially expressed genes (DEGs) in Yanbian cattle BSCs treated with 10 mM L‐carnosine.

## Data Availability

The data that support the findings of this study are available from the corresponding author upon reasonable request.
