# Supplementary material for: L‐Carnosine Enhances the Proliferation and Myogenic Differentiation of Yanbian Cattle Skeletal Muscle Satellite Cells for Cultured Meat Production via Activating the Akt/mTOR/P70S6K Signaling Pathway
Source: Food Sci Nutr. 2026 Apr 24;14(4):e71784. doi: 10.1002/fsn3.71784 (PMC13107544; doi:10.1002/fsn3.71784)
Supplement: Supplementary file 1 — Table S1: Top 10 significantly upregulated and downregulated differentially expressed genes (DEGs) in Yanbian cattle BSCs treated with 10 mM L‐carnosine. [file FSN3-14-e71784-s001.docx]

| ****Gene Symbol**** | ****Gene ID**** | ****log2​FC**** | ****FDR****  ****(padj)**** | ****Regulation**** | ****Gene Description / Function**** |
| --- | --- | --- | --- | --- | --- |
| ***MYH8*** | gene-MYH8 | 4.79 | 1.43E-134 | Up | Myosin heavy chain 8, skeletal muscle |
| ***SCD*** | gene-SCD | 2.55 | 2.52E-134 | Up | Stearoyl-CoA desaturase |
| ***NEB*** | gene-NEB | 2.10 | 6.39E-122 | Up | Nebulin, skeletal muscle giant structural protein |
| ***MYBPC1*** | gene-MYBPC1 | 3.73 | 1.66E-109 | Up | Myosin binding protein C, slow type |
| ***MYL1*** | gene-MYL1 | 2.57 | 1.14E-77 | Up | Myosin light chain 1, skeletal muscle |
| ***ATP1B4*** | gene-ATP1B4 | 3.69 | 1.68E-77 | Up | ATPase Na+/K+ transporting family member beta 4 |
| ***ATP2A1*** | gene-ATP2A1 | 2.72 | 1.01E-75 | Up | SERCA1, fast-twitch skeletal muscle calcium ATPase |
| ***C1QTNF3*** | gene-C1QTNF3 | 2.95 | 1.12E-73 | Up | C1q and TNF related 3 |
| ***ACTA1*** | gene-ACTA1 | 2.28 | 9.88E-72 | Up | Actin alpha 1, skeletal muscle |
| ***PDE4DIP*** | gene-PDE4DIP | 2.09 | 2.23E-69 | Up | Phosphodiesterase 4D interacting protein |
| ***WARS*** | gene-WARS | -2.67 | 7.25E-73 | Down | Tryptophanyl-tRNA synthetase |
| ***PSAT1*** | gene-PSAT1 | -2.45 | 8.40E-65 | Down | Phosphoserine aminotransferase 1 |
| ***FTL*** | gene-FTL | -2.19 | 1.91E-55 | Down | Ferritin light chain |
| ***TIMP3*** | gene-TIMP3 | -2.10 | 6.98E-55 | Down | TIMP metallopeptidase inhibitor 3 |
| ***HSPA9*** | gene-HSPA9 | -1.68 | 7.44E-40 | Down | Heat shock protein family A member 9 |
| ***TXNRD1*** | gene-TXNRD1 | -1.96 | 9.51E-39 | Down | Thioredoxin reductase 1 |
| ***ALDH1L2*** | gene-ALDH1L2 | -2.07 | 3.20E-33 | Down | Aldehyde dehydrogenase 1 family member L2 |
| ***HSPA5*** | gene-HSPA5 | -1.17 | 1.05E-24 | Down | Heat shock protein family A member 5 |
| ***TMBIM6*** | gene-TMBIM6 | -1.23 | 1.40E-24 | Down | Transmembrane BAX inhibitor motif containing 6 |
| ***PSPH*** | gene-PSPH | -2.70 | 4.34E-24 | Down | Phosphoserine phosphatase |

Supplementary Table S1. Top 10 significantly upregulated and downregulated differentially expressed genes (DEGs) in Yanbian cattle BSCs treated with 10 mM L-carnosine.
